# Supporting the ambulance service to safely convey fewer patients to hospital by developing a risk prediction tool: Risk of Adverse Outcomes after a Suspected Seizure (RADOSS)—protocol for the mixed-methods observational RADOSS project

**DOI:** 10.1136/bmjopen-2022-069156

**Published:** 2022-11-14

**Authors:** Adam J Noble, Suzanne M Mason, Laura J Bonnett, Markus Reuber, Jasmine Wright, Richard Pilbery, Richard M Jacques, Rebecca M Simpson, Richard Campbell, Alison Fuller, Anthony Guy Marson, Jon Mark Dickson

**Affiliations:** 1 Department of Public Health, Policy and Systems, University of Liverpool, Liverpool, UK; 2 School of Health and Related Research, The University of Sheffield, Sheffield, UK; 3 Department of Health Data Science, University of Liverpool, Liverpool, UK; 4 Academic Neurology Unit, The University of Sheffield, Sheffield, UK; 5 Public Contributor, UK; 6 Research and Development Department, Yorkshire Ambulance Service NHS Trust, Wakefield, UK; 7 Epilepsy Action, London, UK; 8 Department of Molecular and Clinical Pharmacology, University of Liverpool, Liverpool, UK; 9 Academic Unit of Primary Medical Care, The University of Sheffield, Sheffield, UK

**Keywords:** ACCIDENT & EMERGENCY MEDICINE, Protocols & guidelines, Epilepsy, QUALITATIVE RESEARCH, Health informatics

## Abstract

**Introduction:**

Ambulances services are asked to further reduce avoidable conveyances to emergency departments (EDs). Risk of Adverse Outcomes after a Suspected Seizure seeks to support this by: (1) clarifying the risks of conveyance and non-conveyance, and (2) developing a risk prediction tool for clinicians to use ‘on scene’ to estimate the benefits an individual would receive if conveyed to ED and risks if not.

**Methods and analysis:**

Mixed-methods, multi-work package (WP) project. For WP1 and WP2 we shall use an existing linked data set that tracks urgent and emergency care (UEC) use of persons served by one English regional ambulance service. Risk tools are specific to clinical scenarios. We shall use suspected seizures in adults as an exemplar.

*WP1*: Form a cohort of patients cared for a seizure by the service during 2019/2020. It, and nested Knowledge Exchange workshops with clinicians and service users, will allow us to: determine the proportions following conveyance and non-conveyance that die and/or recontact UEC system within 3 (/30) days; quantify the proportion of conveyed incidents resulting in ‘avoidable ED attendances’ (AA); optimise risk tool development; and develop statistical models that, using information available ‘on scene’, predict the risk of death/recontact with the UEC system within 3 (/30) days and the likelihood of an attendance at ED resulting in an AA.

*WP2*: Form a cohort of patients cared for a seizure during 2021/2022 to ‘temporally’ validate the WP1 predictive models.

*WP3*: Complete the ‘next steps’ workshops with stakeholders. Using nominal group techniques, finalise plans to develop the risk tool for clinical use and its evaluation.

**Ethics and dissemination:**

WP1a and WP2 will be conducted under database ethical approval (IRAS 307353) and Confidentiality Advisory Group (22/CAG/0019) approval. WP1b and WP3 have approval from the University of Liverpool Central Research Ethics Committee (11450). We shall engage in proactive dissemination and knowledge mobilisation to share findings with stakeholders and maximise evidence usage.

**STRENGTHS AND LIMITATIONS OF THIS STUDY**

- Risk of Adverse Outcomes after a Suspected Seizure will use a ‘cutting-edge’ linked data set that captures service use in one ambulance region using data high in quality and coverage.
- The parameters of the outcome measures used to describe risks and the variables tested for their ability to predict these outcomes will be informed by stakeholders and service users.
- The large, pseudoanonymised nature of the linked data set will require the use of a generic definition of an ‘avoidable attendance’ whose validity for suspected seizures is not yet known.
- As there are no equivalent linked data sets available for other ambulance regions, the validity of the derived prediction models will need to be determined within a cohort of patients treated within the same region, but at a later date.

## Introduction

### Context and drive for health service innovations

Ensuring people ‘get the right care at the right time in the optimal care setting’^1^ is a key ambition of the UK’s National Health Service (NHS). Ambulance services have a role to play. They should only be conveying patients to emergency departments (EDs) if it is clinically appropriate or there is no alternative service to provide safe and ongoing care.

Traditionally, UK ambulance services’ primary roles were to provide emergency call handling and transportation to hospital. However, as the nature of the calls it receives has shifted towards non-life-threatening conditions,^2^ services and the clinicians working within them have needed to evolve.^3^

NHS England and Improvement has identified that ambulance clinicians require more support with their changing role.^4^ Certain presentations continue to be ‘over-conveyed’^5^ and reductions in ambulance conveyance rates are stalling.^6^ At the same time, ambulance services are under pressure to provide a timely response to an increasing number of calls,^7^ while facing increased handover delays at EDs.^8^

Strategies are thus needed to support appropriate and safe decision-making on scene that minimises avoidable conveyance. The Risk of Adverse Outcomes after a Suspected Seizure (RADOSS) project seeks to generate ways of providing ambulance clinicians with this support.

### Why is reducing clinically unnecessary conveyance important?

Clinically unnecessary conveyances to EDs result in ‘avoidable attendances’ (AAs).^9^ An AA is where the patient does not require the facilities of a type 1 ED to manage their healthcare problem. AAs can harm the patient^10^ and have implications for others since they restrict ED capacity.^11 12^ Approximately 15% of ED attendances currently meet O’Keeffe *et al*’s^9^ definition of an AA. In 2021/2022, this equated to ~2.3 million visits.^13^

Patients and the public are broadly supportive of non-conveyance. Research has identified that they are frustrated by inappropriate conveyance to ED and say assessment by an ambulance clinician itself has a therapeutic value.^14–21^

Importantly, UK data indicate non-conveyance following assessment by ambulance clinicians is safe. Overall, 83% of people experience no subsequent health event within 3 days of non-conveyance (9% recontact the ambulance service, 12.6% attend ED, 6.3% are admitted and 0.3% die).^22^

### What is known about how ambulance clinicians decide who to convey?

Systematic reviews^23 24^ highlight the complex nature of conveyance decisions. Factors beyond patient need can affect them. Oosterwold *et al*’s^24^ framework (online supplemental file 1) summarises macro, meso and micro factors. Work has started to address some of these.^10 25 26^ However, given reductions in conveyance have stalled, other factors in the model need addressing.

10.1136/bmjopen-2022-069156.supp1 Supplementary data

One factor which has yet to be addressed is that ambulance clinicians can find it difficult to confidently identify cases suitable for non-conveyance. Some report uncertainty regarding the accuracy of their assessments for non-conveyance, and express concern for patient safety and their liability if an incorrect decision is made.^10 27–37^

Their uncertainty is unsurprising. Paramedic education has traditionally focused on life-threatening conditions and transportation; decisions are based on limited clinical information and occur under time pressures. These circumstances can create ‘disproportionate risk aversion’, with patients being conveyed to ED as a precaution or in order to save time.^32 37 38^

### What could help clinicians identify cases suitable for non-conveyance?

Ambulance clinicians are critical of current support, saying non-conveyance guidelines and protocols are difficult to apply to the nuances of cases.^31 33 38 39^ When asked what would help, clinicians identify the development of tools to help them differentiate the needs of individuals as a priority and say the relative risks of non-conveyance for different presentations have also not been fully determined.^10 38 40 41^

Given this, promising ways of supporting clinicians include: (1) securing and disseminating clear evidence on the risks of conveyance and non-conveyance by presentation, and (2) providing a risk prediction tool that would allow clinicians to predict the likelihood that conveyance to ED of the individual they are caring for would result in an AA and the likelihood of them experiencing adverse health events if not conveyed. This direction aligns with recommendations by Lord Carter^42^ and others.^4 26^

### What is a risk prediction tool?

Risk prediction tools use ≥2 pieces of patient data to generate a *personalised* estimate of the likelihood that an individual will experience a certain endpoint within a specified time frame. Currently, there are no prediction tools relating to non-conveyance.^26^ However, evidence suggests they could be developed (see Evidence suggesting a tool predicting benefit/risk of non-conveyance could be developed).

Ambulance clinicians already use such tools to predict other outcomes (eg, ref 43–45) and they want more.^40 46^ The National Ambulance guidelines^47^ currently recommend 11 such tools (none relate to seizures). Risk prediction tools do not replace clinical judgement but support it. There is evidence they can improve patient outcomes and satisfaction and avoid unnecessary care.^48–53^

Methodological standards exist^54^ for their development. To facilitate uptake and sustained use, their development needs to be carefully informed by the views of intended users.^55 56^ There is no single pathway by which a tool enters practice, but good practice states confirmation be obtained that it provides valid predictions on a sample different (in time or place) from the one used for model derivation.^57^

### Evidence suggesting a tool predicting benefit/risk of non-conveyance could be developed

The information used by any risk prediction tool should reflect what is available to the clinician at the time conveyancing decisions are made (and is accessible for derivation). Ambulance clinicians do not typically have access to a patient’s full medical record. What is available is the information they record using structured fields on a patient care record (PCR) about the patient’s demographics, medical history, clinical features, physiological observations as well as details relating to the care provided. Also available is structured dispatch information. Online supplemental file 2 indicates the range of data available.

10.1136/bmjopen-2022-069156.supp2 Supplementary data

So far, only a selection of this information has been examined for its relationship to the outcomes of interest. While exploratory in nature, studies have identified that recontact with the urgent and emergency care (UEC) system and death following non-conveyance, and AAs following conveyance, are not random but more common in certain subgroups (eg, patient age, sex, time of call, day of week, presence of comorbidities and social deprivation^5 9 22 58–60^).

A testament to the utility of the information available to ambulance clinicians are Patton and Thakore’s 61 study findings. ED clinicians reviewed ambulance PCRs of patients conveyed to ED and identified those whose attendances they suspected would be AA. This was then repeated when ED clinicians had access to the PCR data *and* ED notes. Clinicians were confident in identifying AAs on the basis of the PCR alone.

### Current project

#### Overview and aims

To address the identified needs and information gaps, the 24-month mixed-methods RADOSS project is being completed. It has the following aims: (1) calculate the risks and benefits of conveyance to hospital after a suspected seizure; (2) create a risk prediction tool that predicts the likelihood that an individual will die and/or recontact the UEC system within 3 (and 30) days if not conveyed and the likelihood that their conveyance to ED would result in an AA; and (3) establish a pathway to clinical implementation of the risk prediction tool and maximise usage of RADOSS findings. The project’s related objectives were noted in table 1.

**Table 1:** The RADOSS project’s aims and objectives and the work packages that address them

Aims	Objectives
(1) Calculate the risks and benefits of conveyance to hospital after a suspected seizure.	a. Describe the characteristics of those conveyed and those not conveyed to ED by one representative English ambulance service (WP1a).
	b. Compare the proportions following conveyance and non-conveyance that die and/or recontact the UEC within 3 (and 30) days (WP1a).
	c. Quantify the proportion of incidents conveyed to ED that meet the definition of an AA (WP1a).
(2) Create a risk prediction tool that predicts the likelihood that an individual will die and/or recontact the UEC system within 3 (and 30) days if not conveyed and the likelihood that their conveyance to ED would result in an AA.	d. Optimise the prediction tool development by completing KE workshops with service users and ambulance and ED clinicians to get views on predictors considered for inclusion in the models, the way the outcome measures of death, UEC recontact and AA are defined and risk score presentation (WP1b).
	e. Develop statistical models to predict a person’s risk of death/recontact with the UEC system within 3 (and 30) days and the likelihood of their attendance at ED being classed an AA if conveyed (WP1a).
	f. ‘Temporally’ validate the predictive models using data from the same ambulance service for a later time period (WP2).
(3) Establish a pathway to clinical implementation of the risk prediction tool and maximise usage of RADOSS findings.	g. Complete ‘next steps’ workshops with stakeholders to finalise plans to refine the tool for clinical use and its evaluation (WP3).
	h. Complete a proactive dissemination and knowledge mobilisation strategy (WP3, WP4).

AA, avoidable attendance; ED, emergency department; KE, Knowledge Exchange; RADOSS, Risk of Adverse Outcomes after a Suspected Seizure; UEC, urgent and emergency care; WP, work package.

Risk prediction tools are specific to clinical scenarios. We are therefore focusing on patients experiencing suspected seizures. Seizures are a topic of interest in their own right but also an ideal exemplar since they are frequently encountered by the service^62 63^ and ‘over-conveyed’.^10^ Table 2 expands on the reasons.

**Table 2:** Reasons why suspected seizures are considered an ideal exemplar

Reason		Detail
1	Frequently seen	Responsible for ~211 000 ‘999’ calls per year in England; 7th most common presentation.^62 63^ Almost all (>97%) receive a face-to-face ambulance response.^103^
2	‘Over-conveyed’	Around 70% of suspected seizure cases are conveyed to ED.^63 103–106^ This is despite national guidelines stating most will not require ED.^47^ Suspected seizures are dramatic and frightening and traditional training emphasises status epilepticus—a rare and life-threating condition. However, most ‘999’ suspected seizures are low risk and persons return to their normal self without intervention.^63^ Most of those presenting have established, treated epilepsy and have experienced an uncomplicated seizure for which they require rest and reassurance.^107^ Seizures currently have the third highest conveyance rate of all presentations.^10^
3	Redeemable cause of avoidable attendance	Clinicians identify suspected seizures as a readily redeemable cause of AAs.^32 33 35 46 108^ At a 2016 International League Against Epilepsy run research priority event, clinicians identified developing a risk tool to support conveyance decisions as a priority.^46^
4	*‘*Alternative care pathways’ available	Alternative care pathways are becoming available for clinicians to use.^109 110^ They could, unlike visits to ED,^111^ prompt improvements in ambulatory care and so address health inequalities.^112^ Their success depends on clinicians identifying people for them.^113^
5	User preference	People with established epilepsy and those with other seizure presentations, such as non-epileptic attack disorder, usually want to avoid ED after an uncomplicated seizure,^114–117^ preferring to recover at home. Unnecessary conveyance to ED puts them at risk of iatrogenic harm and overinvestigation.^118–120^
6	Cost	Clinically unnecessary ED conveyance generates avoidable costs and contributes to ED overcrowding. In England, the annual cost to the NHS of unplanned hospital care for suspected seizures is ~£90 million.^121^

‘999’ is a telephone number for emergency calls in the UK.

AA, avoidable attendance; ED, emergency department; NHS, National Health Service.

**Figure 1:**
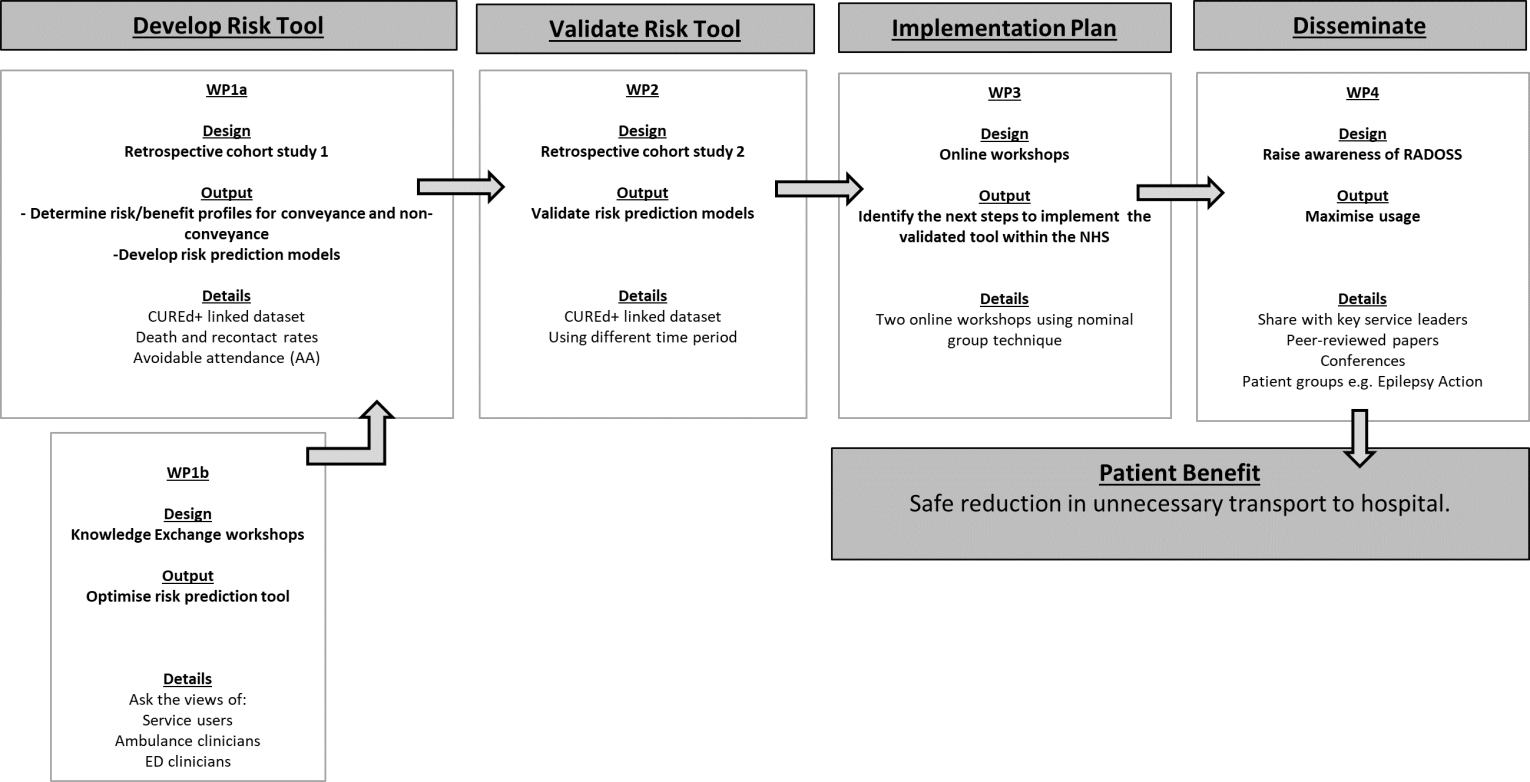
Summary of RADOSS project. Using four WPs, we will develop a risk prediction tool for people after a suspected seizure; we will validate the tool, plan its implementation and disseminate the findings. ED, emergency department; NHS, National Health Service; RADOSS, Risk of Adverse Outcomes after a Suspected Seizure; WP, work package.

RADOSS consists of four work packages (WP). WP1 is the main one. It involves a cohort study (WP1a) and a Knowledge Exchange (KE) study (WP1b). WP2 is smaller and focuses on validation via a second cohort study. WP3 focuses on ‘next steps’ on the journey to implementation of the tool in the NHS, and WP4 on dissemination (figure 1). According to Greene *et al*’s^64^ conceptual framework, the purpose of using a mixed-methods approach is both ‘development’ and ‘expansion’.

### Routine data source: cured+

For WP1a and WP2, we will use a cutting-edge database called ‘CUREd+’. Currently being developed by the Centre for Urgent and Emergency Care Research,^64^ it will map UEC use by individuals served by the Yorkshire Ambulance Service (YAS) from 2011 to 2022. It contains records of all ambulance contacts and these are linkable to any subsequent ambulance, hospital (ED, inpatient) and death records (Office for National Statistics (ONS) mortality register). Further information is provided in table 3.

**Table 3:** Key information about CUREd+ linked database

Issue	Detail
Linkage	CUREd+ is a prelinked data set. Events have been linked by NHS Digital using their algorithm based on NHS number, date of birth, postcode and sex.
Coverage of data	CUREd+ is new. Evidence from its predecessor CUREd (which mapped activity in the same area from 2011 to 2017 using a different approach^122^) indicates CUREd+ should have high data coverage. Evidence from CUREd and other work shows ~85% of individuals can have their ambulance and onward care records linked and so are suitable for inclusion.^104 123 124^ Wider work also shows those who can and cannot have their records linked do not markedly differ.^22^ CUREd+ could have even higher coverage due to more use of NHS numbers by ambulance services^125^ (which supports linkage).
Quality of data	The data contributing to CUREd were high in quality.^121 125 126^ Smyth^76^ examined ambulance patient care records (PCRs) for >22 000 patients. Most core clinical variables had <2% missing data and errors were rare. CUREd+ could have even higher quality due to the introduction of electronic PCRs (which support more consistent data capture).
Area covered by CUREd+ and suitability for RADOSS	England has 10 regional ambulance services. CUREd+ includes data from the Yorkshire Ambulance Service (YAS). The size of the population (~5.6 million) and geographical area (urban/rural mix, ~6000 square miles) served by YAS is similar to the average, as is its non-conveyance rate.^33^

NHS, National Health Service; RADOSS, Risk of Adverse Outcomes after a Suspected Seizure.

## Methods and analysis

### Work package 1

#### WP1a: retrospective cohort study 1

##### Purpose

- Describe the pattern of calls for suspected seizure, the type of ambulance responses received and the characteristics of the patients accounting for them.
- Determine and compare the rate of death and recontact with the UEC system of those seen by the ambulance service for a suspected seizure who were and were not conveyed to ED.
- Determine the proportion of suspected seizure incidents conveyed to ED that resulted in an AA; develop predictive models for risk of death/recontact with the UEC system within 3 (and 30) days following conveyance and non-conveyance and risk of attendance at ED being classed an AA if conveyed.
- Combine the predictive models to form a draft tool that can potentially provide estimates of an individual’s risk of death/recontact with the UEC system if managed by non-conveyance; risk of death/recontact with the UEC system if managed by conveyance; and the risk of their attendance at ED being classed as an AA if conveyed.

To do this, a retrospective cohort of adults cared for a suspected seizure by YAS will be studied.

##### Identification

Index events will be identified by searching CUREd+ for persons managed by YAS for a suspected seizure between 1 February 2019 and 31 January 2020. Eligibility criteria are presented in table 4.

**Table 4:** Participant inclusion and exclusion criteria for different WPs

WP	Inclusion criteria	Exclusion criteria
**WP1a: retrospective cohort study 1**		
	Incident cared for by YAS. Person aged ≥16 years (no upper age limit). (Those ≥16 account for 90% of incidents.^62^ Incident occurred between 1 February 2019 and 31 January 2020.* ‘Chief complaint’ (or other complaint) selected by attending ambulance clinician on PCR was ‘convulsions/fitting/seizure’ OR, if empty, call handler coded it as AMPDS protocol 12 (‘Convulsions’). No restriction on type of ambulance response incident received (ie, could have been ‘Hear & Treat’, ‘See & Treat’, ‘See & Convey to ED’ or ‘See & Convey elsewhere’†).	The <1% of events coded as AMPDSC02. These relate to seizures in someone potentially pregnant. (Guidelines state these should be conveyed because of eclampsia risk.^47^
**WP1b: Knowledge Exchange (KE) workshops**		
Service users	Aged ≥18 years (no upper age limit). Attended to by an ambulance during prior 12 months for a suspected seizure/s OR a significant other to such a person (eg, family member, friend). Incident could be related to epilepsy, non-epileptic attack disorder or syncope. (They account for ~70% of events.^127^ Able to provide informed consent and participate in a workshop independently in English. Lives in England.	Severe current psychiatric disorders (eg, acute psychosis). Terminal medical condition.
Clinicians	Aged ≥18 years (no upper limit). Ambulance clinician, ED doctor or nurse. Works in England. Able to provide informed consent and participate in a workshop independently in English.	Severe current psychiatric disorders (eg, acute psychosis). Terminal medical condition.
**WP2: retrospective cohort study 2**		
	Incident cared for by YAS. Person aged ≥16 years (no upper age limit). Incident occurred between 1 July 2021 and 30 June 2022.‡ ‘Chief complaint’ (or other complaint) selected by attending ambulance clinician on PCR was ‘convulsions/fitting/seizure’ OR, if field was empty, call handler coded it as AMPDS 12. No restriction on type of ambulance response incident received.	The <1% of events coded as AMPDSC02. These relate to seizures in someone potentially pregnant.
**WP3: ‘Next Steps’ workshops**		
	Aged ≥18 years (no upper limit). Ambulance clinician, seizure/epilepsy guideline developer, user group representative, seizure specialist (eg, neurologist/epilepsy nurse), commissioning representative. Able to provide informed consent and participate in a workshop independently in English. Lives in UK.	Severe current psychiatric disorders (eg, acute psychosis). Terminal medical condition.

*The time period does not include periods of industrial action; is before changes in use of acute services due to COVID-19 became apparent^128^; and is before the first UK COVID-19 fatality.^129^

†These are the labels used by the National Health Service (NHS) to record the main types of responses that ambulance services provide to incidents. Further detail is available from NHS England.^130^

‡This time period represents the most contemporary 12 months for which linked data will be available. It excludes COVID-19 national ‘lockdowns’ and the start aligns with when most COVID-19 legal restrictions in England were removed.

AMPDS, Advanced Medical Priority Dispatch System; ED, emergency department; PCR, patient care record; WP, work package; YAS, Yorkshire Ambulance Service.

The unit of analysis will be the patient, with the first recorded episode being the index event and subsequent episodes ≤3 days defined as recontacts (or 30 days for the secondary analysis).

##### Data extract

The data extract provided will include any ambulance, ED (Emergency Care Data Set; Hospital Episode Statistics (HES) Accident and Emergency (due to overlap in system use)), urgent inpatient (HES Admitted Patient) and death (ONS) records that relate to the index events which started within 30 days.

##### Outcome measures

Death/recontact with the UEC system following ambulance care and the likelihood of an AA occurring if conveyed to ED are important outcomes to clinicians and service users.^65^ Below we describe how the index events will be classified according to these two measures.

###### Measure 1 (safe/unsafe: death or recontact with UEC)

All index events, both conveyed and non-conveyed to ED, will be classified according to whether linked data indicate the patient involved died and/or recontacted the UEC (defined as any ambulance, ED or unscheduled inpatient care).

For the primary analysis, we propose a time frame of up to 3 days from the event within which death must have occurred or recontact started. This has been specified by paramedics and other stakeholders.^66^ It aligns with evidence that when considering *all* ambulance presentations, ~75% of deaths/UEC recontacts following non-conveyance occur within 3 days.^22^ We shall though still confirm its suitability with clinicians and service users via WP1b. For secondary analyses, a time frame of 30 days is proposed.^37 66–69^

Deaths within the cohort should be rare. Nonetheless, when describing and using deaths we shall report them with and without exclusion of persons where death was associated with end-of-life care.

###### Measure 2 (avoidable/unavoidable ED attendance)

Index events that resulted in conveyance to ED will be classified according to whether they resulted in an AA or not.

To determine this, the events will be assessed against O’Keeffe *et al*’s^9^ definition. Namely, a person has been involved in an AA if routine hospital coding for the attendance indicates it did not result in the person being investigated (except urinalysis, pregnancy test, dental investigation) or treated (except prescription, recording vital signs, dental treatment or guidance/advice), and they were discharged.

O’Keeffe’s system has advantages. It is generic, applicable to all ages,^9 70^ based on process of care rather than initial triage score and has been adopted by the NHS.^71^ It is also quick and routine data have been found to be sufficient to mean it can be applied to ~98% of attendances.^9^

A possible disadvantage is it assumes *all* investigations, treatments and admissions were clinically indicated. Some may have happened for other reasons (eg, routine or inappropriate administration of test). Thus, we shall describe the reasons why any WP1a cases satisfied the criteria for an unavoidable attendance. Moreover, via WP1b, we shall ask ED clinicians to what extent suspected seizure cases attending their EDs could satisfy the criteria of an unavoidable attendance based on routine practice. Should it prove warranted, a sensitivity analysis will be conducted with and without such cases.

##### Sample size

Predictive models for the (1) risk of death/UEC recontact following conveyance, (2) risk of death/UEC recontact following non-conveyance, and (3) risk of an AA following conveyance could be developed. To permit robust testing of at least 40 candidate predictor parameters for each of these models, Riley *et al*’s^72^ formulae using standard parameters indicate: for model (1), a need for 2567 index events, with 103 experiencing the target event; for model (2), a need for 2194 index events, with 308 experiencing the target event; and for model (3), up to 2194 index events, with 461 experiencing the target event. Twelve months of YAS data should be sufficient to satisfy these requirements. Online supplemental file 3 details the reasons why and provides further information on the sample size calculation.

10.1136/bmjopen-2022-069156.supp3 Supplementary data

##### Data management and analysis

###### Curation

A statistician, with support from a data manager, will complete data quality checks on the data extract, identifying missing and incongruent values.

###### Describing sample and patient outcomes

The characteristics of the calls for suspected seizures (dispatch codes, time of day, day of week, location), the patients accounting for them and the ambulance response they receive (proportions managed by ‘Hear & Treat’, ‘See & Treat’, ‘See & Convey to ED’ and ‘See & Convey elsewhere’) will be described.

For events receiving the response ‘See & Convey to ED’, we shall:

- Tabulate ED discharge diagnoses.
- Calculate the proportion satisfying the AA definition.
- Tabulate the reason/s why persons did not satisfy the AA definition.
- Calculate the proportion recontacting the UEC system within 3 (and 30) days (with and without inclusion of those whose subsequent contact/s meet the AA definition).
- Calculate the proportion dying within 3 (and 30) days and reasons.

For events receiving a face-to-face response but not conveyed to ED (ie, ‘See & Treat’, ‘See & Convey elsewhere’), we shall:

- Calculate the proportion recontacting UEC system within 3 (and 30) days (with and without those whose subsequent contact/s meet the AA definition; also, with and without those originally non-conveyed to ED because they refused).^14 73^
- Calculate the proportion dying within 3 (and 30) days and reasons.

###### Derivation of prediction models and management of missing data

As the outcome measures are binary, multivariable logistic regression will be used to derive the predictive models.^57^ Reporting will be done according to best practice.^74^ The pool of candidate predictors for testing will be informed by WP1b (see WP1b: KE workshops) and chosen based on clinical relevance, consistency in measurement and ease of use in practice.^75^ Where possible, variables will be used in their original form.

While missingness on core data items is anticipated to be low,^62 76^ missingness on wider items might be higher since tests may not be performed if expected to be normal and not all PCR fields are mandatory.^77 78^ Where data are ‘expectedly’ missing (ie, the test is not performed as not clinically indicated), an additional category of ‘not clinically indicated’ will be added to the variable. In the case of more than 10% missingness for any other variable, multiple imputation via chained equations will be undertaken. A set of 20 imputed data sets will be created using predictive mean matching.^79^ Functional form for continuous variables will be assessed via fractional polynomials within each imputed data set.^80^ Variables will be selected for inclusion in the final model within each imputed data set via backward selection with a p value of 0.10. Variables that feature in at least 10 of the 20 imputed models will be selected for the final model. Pooled OR and intercepts will be calculated according to Rubin’s rule.

Apparent measures of model performance will be calculated for the final multiple imputed model. The area under the receiver operating characteristic curve will be calculated to assess the final model’s discriminative performance. Discrimination refers to the ability of the prognostic model to differentiate between those who experienced the event and those who did not. We will report the calibration slope and the ratio of expected to observed events to evaluate calibration, how closely the probability of the event predicted by the model agrees with the observed probability. C-statistics resulting from the imputed data set will be pooled via robust methods and therefore the median of the imputed estimates will be presented.^81 82^ Calibration will also be observed via a calibration plot for each imputed data set separately and the median of the imputed estimates provided.^82^

To account for sampling variability and enable adjustment of the regression coefficients for overfitting,^83^ the final model will be internally validated via bootstrap resampling. In each of 500 bootstrap samples, the entire modelling process, including predictor selection, will be repeated and the apparent model performance (calibration and discrimination in the bootstrap sample) will be compared with the performance in the original sample per multiple imputed data set. The median optimism across all imputed samples will then be used to calculate the optimism-adjusted C-statistic and optimism-adjusted calibration slope.^84^ Using the latter as a uniform shrinkage factor, all the predictor effects in the final developed model will be penalised in order to account for overfitting.^85^

The pool of potential predictors for the backward selection will be any predictor in a final multivariable model for each imputed data set.

###### Combining the predictive models to form a draft tool

The three derived models will be combined to form a single, Excel-based draft version of a tool that seeks to provide estimates of an individual’s risk of death/recontact with the UEC system if managed by non-conveyance; risk of death/recontact with the UEC system if managed by conveyance; and the risk of their attendance at ED being classed as an AA if conveyed. The manner in which it is presented will be informed by WP1b and previous work by Bonnett *et al*.^86^ Examples of tools that have combined predictive models to provide clinicians with different estimates to inform decisions include the CHA2DS2-VASc/HAS-BLED^87^ and the cancer PREDICT tool.^88^

#### WP1b: Ke workshops

##### Purpose

- Optimise prediction tool development by completing KE workshops with service users, ambulance clinicians and ED clinicians to get views on candidate predictors, the way the outcome measures of death, UEC recontact and AA are defined and risk score presentation.

##### Design

KE workshops will be run online using videoconferencing technology. Wilkins and Cooper^89^ defined KE as a two-way exchange between researchers and research ‘users’ to share ideas, evidence, experiences and skills. It goes beyond telling people things and is a process of listening and interaction, with a goal to generate mutual benefit.

##### Participants

###### Service users

Purposive sample of ~20–30 persons recently receiving ambulance care for a suspected seizure/s and their significant others. Full eligibility is presented in table 4.

Individuals shall be recruited via user groups affiliated with the different conditions (including epilepsy deaths). They shall circulate advertisements directly to their members and within publications.

###### UEC clinicians

Sampling will be purposive, consisting of a group of ~20–30 informed individuals/‘experts’ deemed to have high professional knowledge and clinical experience of the UEC system.

The national ‘Lead Paramedic Group’ will circulate advertisements, with priority being given to ambulance clinicians from the n=6 services that have used Advanced Medical Priority Dispatch System. To recruit ED clinicians, the Royal College of Emergency Medicine Yorkshire and Humber regional board shall circulate advertisements.

##### Procedure

Workshops for service users and ambulance clinicians will run separately. To maximise participation, we anticipate two to three for each. They will be conducted by a qualitative researcher. For those with clinicians, statistician LJB will assist.

Workshops will start with an explanation of the risk tool, aims and a presentation of the potential predictors and proposed outcome measures. A topic guide will direct the conversation. It will be finalised on the basis of the literature,^86 90^ our experience and key uncertainties regarding the tool’s future implementation surfaced by completion of Greenhalgh *et al*’s Non-adoption, Abandonment, Scale-up, Spread and Sustainability Complexity Assessment Tool Long.^91^ The main areas that the workshops intend to cover are shown in table 5. Workshops will last ~60–90 min.

**Table 5:** Topic guide areas that WP1b Knowledge Exchange workshops will explore (emphasis will vary depending on whom the workshop is for)

Area		Detail
1	Potential predictors	Asked for views on potential predictors, including perceived utility, reliability, validity and consistency in measurement.^131^
2	Parameters of outcome measures	Asked whether any routine ED practices could mean seizure cases by default would not satisfy AA definition. Asked about any known differences between EDs and hospitals in how codes for incidents are applied at them that could undermine validity of definition that is based on them. What time frame for death and recontact with UEC would be most supportive for conveyance decisions.
3	Optimal way to present risk scores	Asked whether percentage probability and/or broad risk categories wanted, whether visual aids would help and if estimates of uncertainty around probabilities wanted. Illustrations of options offered. Asked what ‘low’, ‘medium’ or ‘high’ risk of death, UEC recontact or AA would look like to them in percentage terms.
4	Optimal format for tool	Asked how they might want such a tool to be presented in future (eg, web tool, nomogram, graphical score chart), who should have access to it and the extent to which they would want it integrated into existing workflows.^132^

AA, avoidable attendance; ED, emergency department; UEC, urgent and emergency care; WP, work package.

##### Analysis

Data will include field notes and audio recordings. A qualitative researcher, supported by the wider team, will take an inductive and deductive approach to analysis. NVivo will provide a transparent account of the work. Nodes (codes) will be created to mark relevant concepts and topics in the documents. Lower level nodes will be grouped into themes.

### Work package 2

#### WP2: Retrospective cohort study 2

##### Purpose

- ‘Temporally’ validate WP1a’s predictive models.

The predictions of the WP1a models will be tested on a data set relating to patients cared for by YAS during a 12-month time period different from that used for derivation.

##### Identification, data linkage, data checks and outcome measures

CUREd+ will be searched to identify events as done for WP1a, except the date range will be 1 July 2021 to 30 June 2022 (table 4). Outcome measures and processes used will be the same.

##### Sample size

The validation sample will be similar to that used for derivation. It will thus satisfy the recommendation that validation samples include ≥200 cases experiencing the target events.^92 93^

##### Data management and analysis

###### Describing sample and patient outcomes

Sample contributing data will be described as for WP1a.

###### Comparison with time period used for model derivation

Number of calls for and the characteristics of the patients presenting with suspected seizures during the derivation and validation periods will be compared, as will the proportions conveyed to ED, the proportions whose attendance meets the AA definition and the proportions dying/recontacting the UEC within 3 (and 30) days. Differences will be described and tested for statistical significance.

###### Temporal validation of predictive models

Predictors and regression coefficients from the final internally validated, optimism-adjusted models will be applied to the WP2 data set to predict the target outcomes. The performance of the models will be quantified by comparing predictions with observed outcomes.^94^ Performance will be assessed using measures of discrimination and calibration. Model recalibration will be undertaken if there is systematic underprediction or overprediction.^95^

### Work package 3

#### Next steps’ workshops

##### Purpose

- Finalise plans to refine the risk tool for clinical use and its evaluation.

If the developed models are found to make predictions with an acceptable level of validity then we would have satisfied the requirements for the tools use within practice. We would therefore need to finalise its presentations for clinical use and evaluate its impact on clinical practice. To ensure any plans for this are acceptable to stakeholders and address their information needs, ‘Next steps’ workshops will be completed.

##### Design

Two online workshops, each lasting ~3 hours. We shall limit each to approximately eight to nine participants.^96 97^

Workshops will start with a presentation of RADOSS findings and our draft ‘next steps’. To secure stakeholders’ views of these we would use an adapted version of the nominal group technique.^97^

With respect to what evaluation we propose we consider it appropriate to make this judgement nearer the time. A cluster randomised controlled trial would likely be most rigorous. However, various factors can influence and constrain design choice.^55 98^ This includes time frame within which evidence is required and regulations at the time surrounding risk tools.^99^

##### Recruitment

We shall seek representation from:

- Service providers (via Association of Ambulance Chief Executives National Ambulance Strategy and Transformation Group).
- Care guideline providers (via Joint Royal Colleges Ambulance Liaison Committee panel for seizures; National Institute for Health and Care Excellence panel for epilepsy).
- User groups (including Epilepsy Action, Epilepsy Society, FND Action, SUDEP Action and others).
- Ambulance research and care quality improvement (via National Ambulance Steering Group; National Ambulance Services Clinical Quality Group).
- Seizure specialists (via International League Against Epilepsy; Epilepsy Specialists Nurses Association).
- Commissioners (via National Ambulance Commissioners Network).

Personal invitations will be sent. To maximise attendance, we shall exploit existing relationships our team has. We shall overinvite by ~30%.^100^ Table 4 provides the eligibility criteria.

##### Procedure

Workshops will be facilitated by the investigative team. Presentations will be pre-recorded to reduce opportunity for technical difficulties.

##### Analysis

Field notes will be kept. Delegates’ involvement will be anonymous. A summary of the findings will be generated and discussed by the investigators and the ‘next steps’ plan finalised.

### Work package 4

#### Purpose

- Disseminate findings to key stakeholders and maximise evidence usage.

#### Dissemination and outputs

We shall engage in a proactive dissemination and knowledge mobilisation strategy to ensure those who are considering developing, funding or supporting non-conveyance strategies are aware of the project and its findings. All investigators shall contribute, and the media departments of involved institutions shall help. As well as conducting WP3, dissemination will consist of the items in table 6.

**Table 6:** Dissemination actions (in addition to WP3)

Activity		Detail
1	Promoting awareness/engagement	Notification of the project’s funding and progress sent to medical directors and lead consultant paramedics of all ambulance services, National Clinical Director for Urgent Care for NHS England, National Ambulance Commissioners Network; National Ambulance Urgent and Emergency Care Group subgroup of the Association of Ambulance Chief Executives, National Ambulance Research Steering Group.
2	Interim updates	As project progresses, accessible briefings are produced and disseminated to funders; stakeholders; service user groups; policy makers; NHS audiences; and research bodies. Include NHS Improvement and NICE who identified need for such research.
3	Peer-reviewed outputs	Minimum of 2 papers in peer-reviewed journals which would appeal to clinical, organisational, general health and social policy audiences.
4	Taking evidence to practitioners	Findings circulated via NHS network newsletters, in practitioner journals and general press.
5	Taking evidence to clinicians	Oral and poster presentations at neurology and acute/emergency care conferences and fora.
6	Taking evidence to participants	Summary of project’s findings distributed to participants in the different WPs.
7	Media briefings	Updates on websites including YAS, Epilepsy Action and universities.
8	Taking evidence to service users	Service users and significant others/carers will clearly be interested in study outcomes. Epilepsy Action will feature study with patient experience stories in communications with epilepsy community.

NHS, National Health Service; NICE, National Institute for Health and Care Excellence; WP, work package; YAS, Yorkshire Ambulance Service.

## Discussion

### Patient and public involvement

This research was instigated by evidence on the priorities of the seizure community and those supporting them (eg, ref 101). To shape the project’s design and determine its perceived importance, a patient and public involvement (PPI) event for nine service users and their informal carers was completed. A similar exercise was completed with leading clinicians from seven of England’s ambulance services. Both groups were supportive of the project idea and provided feedback on the project’s draft design. When asked to rate its importance on a scale of 1–10, seven service user pairs scored it as 10 ‘Extremely important’.

Services users will be actively involved in the project’s completion. Service users are present in both the research team and in the groups advising and overseeing it. Coinvestigator JW is a service user herself with experience in ambulance care. Epilepsy Action, the largest seizure user organisation in the UK, is also a coinvestigator. A PPI group of 20 user representatives will contribute as research peers, advising the investigators on recruitment and reviewing study conclusions, implications for practice and recommendations. Four user representatives will also be on RADOSS Study Steering Committee (SSC).

All user representatives will be supported by Epilepsy Action who have an active PPI scheme and reimbursed for travel and their time according to guidance.^102^ Representatives will be recruited from a range of user groups.

### Ethics and dissemination

Monitoring by an independent SSC will help to ensure the rights, safety and well-being of participants are the most important considerations. Compliance with the principles of Good Clinical Practice and scientific integrity will be managed by the study management team through regular and ad hoc meetings. YAS will be the sponsor. AJN and JMD are cochief investigators. WP1a and WP2 will use completely anonymised data from CUREd+. Access will be sought from the Centre for Urgent and Emergency Care Research Data Release Committee. CUREd+ has generic database ethical approval (307353) and Confidentiality Advisory Group approval (22/CAG/0019). With strict controls, WP1a and WP2’s work will be completed under these. WP1b and WP3 have received ethical approval from the University of Liverpool Central Research Ethics Committee D (11450). Only persons providing informed consent will participate.

We shall engage in a proactive dissemination and knowledge mobilisation strategy. It is specifically addressed by WP4 described in section Work package 4.

All requests for data sharing should be submitted to the corresponding author for consideration. Access to anonymised data may be granted following review.

## Supplementary Material

Reviewer comments
