# Supplementary material for: Supporting the ambulance service to safely convey fewer patients to hospital by developing a risk prediction tool: Risk of Adverse Outcomes after a Suspected Seizure (RADOSS)—protocol for the mixed-methods observational RADOSS project
Source: BMJ Open. 2022 Nov 14;12(11):e069156. doi: 10.1136/bmjopen-2022-069156 (PMC9668054; doi:10.1136/bmjopen-2022-069156)
Supplement: Supplementary data [file bmjopen-2022-069156supp001.pdf]

SUPPLEMENTARY FILE 1-

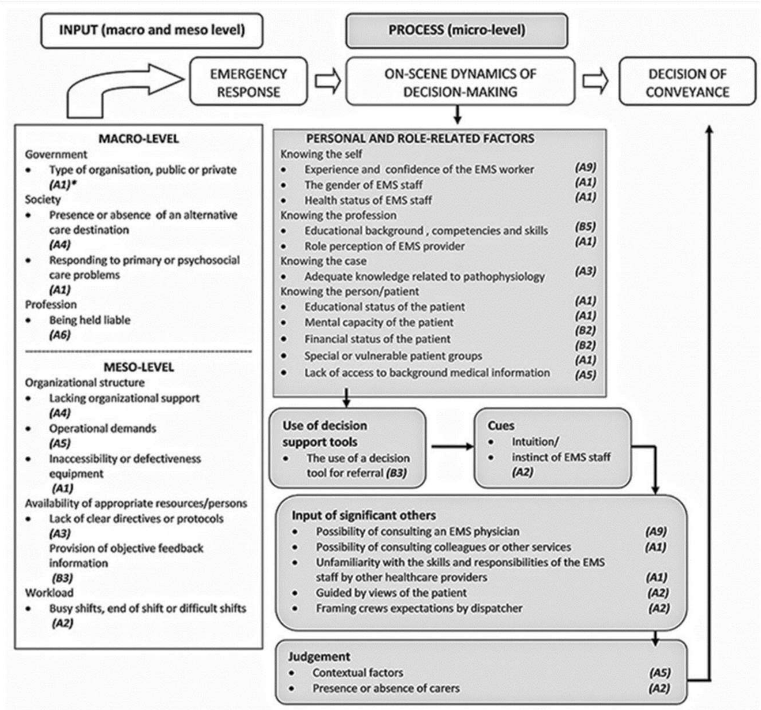

FIGURE S1.1 Conceptual framework of factors affecting ambulance clinicians' conveyance decisions (Oosterwold et al. [1])

Notes: The framework does not assert importance or interplay between factors. It does, though, highlight the quality of evidence support their importance ('A' is highest). That clinicians can find it difficult to confidently identify cases suitable for non-conveyance is captured under 'Experience and confidence of the emergency medical service [EMS] provider' and 'Educational background, competencies and skills' in the figure. Reproduced with permission (5347751335679).

REFERENCES

1. Oosterwold, J., et al., *Factors influencing the decision to convey or not to convey elderly people to the emergency department after emergency ambulance attendance: a systematic mixed studies review.* BMJ Open, 2018. **8**(8): p. e021732.
