# Supplementary material for: Supporting the ambulance service to safely convey fewer patients to hospital by developing a risk prediction tool: Risk of Adverse Outcomes after a Suspected Seizure (RADOSS)—protocol for the mixed-methods observational RADOSS project
Source: BMJ Open. 2022 Nov 14;12(11):e069156. doi: 10.1136/bmjopen-2022-069156 (PMC9668054; doi:10.1136/bmjopen-2022-069156)
Supplement: Supplementary data [file bmjopen-2022-069156supp002.pdf]

SUPPLEMENTARY FILE 2

TABLE S2.1 Range of structured data items that can be available to ambulance clinicians whilst on scene and will be included in data extract for RADOSS index events [a, b]

Data items	Ambulance data system recorded within
• Source of call (999, 111, police, other)	CAD
• Relationship of the caller to the patient	CAD
• Incident Date (day of week and month will be used)	CAD
• Incident Time(s)	CAD/PCR
• Patient age	PCR
• Patient sex	CAD
• Patient ethnicity	CAD
• Chief Complaint	CAD
• Dispatch AMPD code and description [c, d]	CAD
• Priority category for response time	CAD
• Location type (private residence/ public place/ place of work/education)	PCR<br>PCR
• Incident location (Index of Multiple Deprivation 2020 and subdomain decile scores (IMD2020) and Rural Urban Classification of incident location (RUC11CD) will be used) [e]	CAD/PCR
• Presenting complaint	PCR
• Clinician grade	PCR
• Was patient transported	PCR
• Type of facility transported to (ED, UTC, etc.)	PCR
• Reason for non-transport	PCR
• Referral to other service - type	PCR
• Pre-alert to hospital	PCR
• Physiological observations (e.g., pulse, BP, respiratory rate, oxygen saturation, level of consciousness AVPU, GCS, NEWS, blood sugar, temperature,)	PCR
• Previous medical history reported	PCR
• Airway intervention – type	PCR
• Wound care provided	PCR
• ECG findings	PCR
• Cardiac or respiratory arrest present	PCR
• Cardiac or respiratory arrest outcome	PCR
• Advice provided (non-transported patients)	PCR
• Supplementary oxygen provided	PCR
• Current medications	PCR
• Drugs administered (name, dosage, route)	PCR
• Time arrived scene	PCR
• Time departed scene	PCR

Notes: CAD, computer aid dispatch; PCR, patient care record; [a] Professional standards mean clinicians document findings from ≥1 set of observations[1]; [b] The RADOSS team will only have access to anonymised data; For reasons of governance and cost, the data extract for the index events will not

include free-text data entered by clinicians on ePCRs; [c] Like most services, the Yorkshire Ambulance Service use the Advanced Medical Priority System (AMPDS) to code reason for call. When a call is received regarding what is described as a suspected seizure then AMPDS Protocol 12 is activated. It prompts call handlers to ask standard questions about the presentation severity and the patient. The responses inform the specific subcode given to the call. One question asked is whether the patient has a diagnosis of epilepsy (i.e., "Is s/he an epileptic?"); [d] A minority (~5%) [2] of suspected seizure calls are transfers from '111' rather than '999' calls. As these calls were not managed via AMPDS, a slightly different data-set is available for them (e.g, disposition code). It will be included in the RADOSS data-extract. [e] These items will have been calculated based on LSOA codes by the central CURE'd team who have access to pseudoanonymised data..

REFERENCES

1. Joint Royal Colleges Ambulance Liaison Committee/ Association of Ambulance Chief Executives, *JRCALC Clinical Guidelines* 2019, Bridgwater: Class Professional Publishing.
2. Hughes-Gooding, T., et al., *A data linkage study of suspected seizures in the urgent and emergency care system in the UK. .* Emergency Medicine Journal 2020. **10**: p. 605-610.
