# Supplementary material for: Supporting the ambulance service to safely convey fewer patients to hospital by developing a risk prediction tool: Risk of Adverse Outcomes after a Suspected Seizure (RADOSS)—protocol for the mixed-methods observational RADOSS project
Source: BMJ Open. 2022 Nov 14;12(11):e069156. doi: 10.1136/bmjopen-2022-069156 (PMC9668054; doi:10.1136/bmjopen-2022-069156)
Supplement: Supplementary data [file bmjopen-2022-069156supp003.pdf]

SUPPLEMENTARY FILE 3

Details on the size of available linked data set and anticipated number of target events

Twelve-months of Yorkshire Ambulance Service (YAS) data should be sufficient to satisfy the requirements. It should permit a linked data-set to be formed for 13,980 index events [1, 2]. This is after excluding ~15% of cases that might have unlinkable records, ~3% who have 'opted-out' of research use of their data,[3] and the 0.02% of cases where the person died 'on-scene'.[2]

Of the index events, 5,720 index events should have been conveyed of whom 229 (4%) will have experienced death/UEC recontact within 3 days and 1,201 (21%) could have experienced an AA. The remaining 2,451 index events should have been managed by non-conveyance, of whom 343 (14%) will have experienced death/ recontact with the urgent and emergency care system (UEC).[4-6]

The estimate that 14% of events not conveyed to ED will lead to death and/or recontact with the UEC within 3 days is based on O'Cathian et al.'s UK study.[4] We estimated that 4% of cases conveyed to ED will result in death and/or UEC contact. This estimate was generated differently since there is no UK evidence on this. Specifically, we considered Tohira et al.'s[5] US study. It reported adverse event rates within 24 hours in all persons who had attended ED for any reason were 2-4 times lower than for those not conveyed. It was also necessary to factor in individuals accounting for several types of events. Based on evidence from Coster et al.[7] we divided O'Cathian's[4] estimates by 2 to account for this possibility and then applied Tohira et al. ratios to O'Cathian's figures.

The estimate that 21% of seizure cases attending ED would satisfy the AA definition is informed by Miles et al.[6].

Key information factored into sample size calculation using Riley et al.'s formulae for each of the models

1.	Permit testing of ≥40 candidate predictor parameters
2.	Assume 0.05 acceptable difference in apparent and adjusted R-squared
3.	Assume 0.05 margin of error in estimation of intercept
4.	In the absence of other information, conservatively assumed that R^2_{CS} for each model would correspond to an $R^2_{Nagelkerke}$ of 0.15. [8]

REFERENCES

1. Dickson, J.M., et al., *Cross-sectional study of the prehospital management of adult patients with a suspected seizure (EPIC1).* BMJ Open, 2016. **6**(2): p. e010573.
2. Dickson, J.M., Z.B. Asghar, and A.N. Siriwardena, *Pre-hospital ambulance care of patients following a suspected seizure: A cross sectional study.* Seizure, 2018. **57**: p. 38-44.
3. Digital, N., *[MI] National Data Opt-out, March 2019*. 2020.
4. O'Cathain, A., et al., *Understanding variation in ambulance service non-conveyance rates: a mixed methods study.* Health Services and Delivery Research 2018. **6**(19).
5. Tohira, H., et al., *Is it appropriate for patients to be discharged at the scene by paramedics?* Prehospital Emergency Care, 2016. **20**(4): p. 539–549.
6. Miles, J., et al., *Exploring ambulance conveyances to the emergency department: a descriptive analysis of non-urgent transports.* Emergency Medicine Journal, 2017. **34**: p. A872-A873.
7. Coster, J., et al., *Outcomes for Patients Who Contact the Emergency Ambulance Service and Are Not Transported to the Emergency Department: A Data Linkage Study.* Prehospital Emergency Care, 2019. **23**(4): p. 566-577.
8. Riley, R.D., et al., *Calculating the sample size required for developing a clinical prediction model.* British Medical Journal, 2020. **368**: p. m441.
