# Supplementary material for: Supporting the ambulance service to safely convey fewer patients to hospital by developing a risk prediction tool: Risk of Adverse Outcomes after a Suspected Seizure (RADOSS)—protocol for the mixed-methods observational RADOSS project
Source: BMJ Open. 2022 Nov 14;12(11):e069156. doi: 10.1136/bmjopen-2022-069156 (PMC9668054; doi:10.1136/bmjopen-2022-069156)
Supplement: Reviewer comments [file bmjopen-2022-069156.reviewer_comments.pdf]

PEER REVIEW HISTORY

BMJ Open publishes all reviews undertaken for accepted manuscripts. Reviewers are asked to complete a checklist review form (**http://bmjopen.bmj.com/site/about/resources/checklist.pdf**) and are provided with free text boxes to elaborate on their assessment. These free text comments are reproduced below.

ARTICLE DETAILS

**TITLE (PROVISIONAL)**	Supporting the ambulance service to safely convey fewer patients to hospital by developing a risk prediction tool: Risk of ADverse Outcomes after a Suspected Seizure (RADOSS): protocol for the mixed methods observational RADOSS project
**AUTHORS**	Noble, Adam; Mason, Suzanne; Bonnett, Laura J.; Reuber, Markus; Wright, Jasmine; Pilbery, Richard; Jacques, Richard; Simpson, Rebecca; Campbell, Richard; Fuller, Alison; Marson, Anthony; Dickson, Jon

This article was not externally reviewed at BMJ Open. Protocols that have been independently assessed prior to submission to BMJ Open are usually fast-tracked to publication on the grounds that further substantial changes will not be possible. This independent assessment will usually be external, independent review for both a substantial grant award from a non-commercial or government funder and ethics approval.
